# Underlying Histopathology Determines Response to Oxidative Stress in Cultured Human Primary Proximal Tubular Epithelial Cells

**DOI:** 10.3390/ijms21020560

**Published:** 2020-01-15

**Authors:** Muhammad Ali Khan, Xiangju Wang, Kurt T.K. Giuliani, Purba Nag, Anca Grivei, Jacobus Ungerer, Wendy Hoy, Helen Healy, Glenda Gobe, Andrew J. Kassianos

**Affiliations:** 1 NHMRC CKD CRE (CKD.QLD), University of Queensland, Brisbane 4029, Queensland, Australia; Muhammad.A.Khan@uq.edu.au (M.A.K.); W.Hoy@uq.edu.au (W.H.); Helen.Healy@health.qld.gov.au (H.H.); G.Gobe@uq.edu.au (G.G.); 2 Faculty of Medicine, University of Queensland, Brisbane 4006, Queensland, AustraliaJacobus.Ungerer@health.qld.gov.au (J.U.); 3 Conjoint Internal Medicine Laboratory, Chemical Pathology, Pathology Queensland, Brisbane 4029, Queensland, Australia; Xiangju.Wang@qimrberghofer.edu.au (X.W.); Purba.Nag@qimrberghofer.edu.au (P.N.); Anca.Grivei@qimrberghofer.edu.au (A.G.); 4 Kidney Health Service, Royal Brisbane and Women’s Hospital, Brisbane 4029, Queensland, Australia; 5 Kidney Disease Research Collaborative, Princess Alexandra Hospital and University of Queensland, Translational Research Institute, Brisbane 4102, Queensland, Australia; 6 Department of Pharmacy, Bangabandhu Sheikh Mujibur Rahman Science and Technology University, Gopalganj 8100, Dhaka, Bangladesh; 7 Centre for Chronic Disease, Faculty of Medicine, University of Queensland, Brisbane 4029, Queensland, Australia

**Keywords:** human primary proximal tubular epithelial cells, oxidative stress, acute kidney injury, chronic kidney disease

## Abstract

Proximal tubular epithelial cells (PTEC) are key players in the progression of kidney diseases. PTEC studies to date have primarily used mouse models and transformed human PTEC lines. However, the translatability of these models to human kidney disease has been questioned. In this study, we investigated the phenotypic and functional response of human primary PTEC to oxidative stress, an established driver of kidney disease. Furthermore, we examined the functional contribution of the underlying histopathology of the cortical tissue used to generate our PTEC. We demonstrated that human primary PTEC from both histologically ‘normal’ and ‘diseased’ cortical tissue responded to H_2_O_2_-induced oxidative stress with significantly elevated mitochondrial superoxide levels, DNA damage, and significantly decreased proliferation. The functional response of ‘normal’ PTEC to oxidative stress mirrored the reported pathogenesis of human kidney disease, with significantly attenuated mitochondrial function and increased cell death. In contrast, ‘diseased’ PTEC were functionally resistant to oxidative stress, with maintenance of mitochondrial function and cell viability. This selective survival of ‘diseased’ PTEC under oxidizing conditions is reminiscent of the in vivo persistence of maladaptive PTEC following kidney injury. We are now exploring the impact that these differential PTEC responses have in the therapeutic targeting of oxidative stress pathways.

## 1. Introduction

Kidney disease is a major public health problem, affecting approximately 10% of populations in industrialized countries [1]. Most notably, the global burden of kidney disease continues to rise, driven by population growth, ageing, and the increased prevalence of diabetes and hypertension [2]. The development of disease within the kidney is characterized on renal biopsy by microstructural patterns of pathology, including arteriosclerosis, glomerulosclerosis, and tubular atrophy/interstitial fibrosis [3]. Regardless of the diverse etiologies underlying these histopathological changes, oxidative stress is a common and key pathobiological driver of kidney injury [4,5].

Oxidative stress refers to an elevation in levels of intracellular reactive oxygen species (ROS) that initiate damage to lipids, proteins, and nucleic acids [6]. ROS, including mitochondrial superoxide (O_2_^−^), are natural byproducts of aerobic metabolism, and have significant functional roles in intracellular signaling and homeostasis. However, an imbalance between the cellular production of ROS and the capacity of cells to neutralize them results in oxidative stress [7]. In turn, oxidative stress drives mitochondrial dysfunction associated with mitochondrial swelling and the loss of mitochondrial membrane potential (MMP), leading to altered gene expression and cell death via apoptotic or necrotic pathways [5]. 

Oxidative stress disrupts all functional aspects of the kidney, including proximal tubular reabsorption [8]. The epithelial cells of the proximal tubules (PTEC) are a major source of ROS in the kidney due to their high mitochondrial density and the immense energy demands required to support their reabsorption of vital molecules from the glomerular filtrate [4]. As a result, PTEC are particularly susceptible to oxidant-induced damage and death [9]. The maladaptive repair mechanisms of PTEC in response to injury, including oxidative stress, are established as central mediators of kidney disease progression. However, previous studies of oxidative stress in PTEC have focused primarily on mouse models or cell lines, including transformed human PTEC lines [8]. We propose to translate this body of work into human primary PTEC, to examine the pathobiology of oxidant-induced kidney disease in a biological system of physiological relevance to humans. The oxidative stress response of human primary PTEC may, however, be affected by the underlying histopathology of the cortical tissue used to generate these PTEC. This has yet to be investigated.

In this present study, human primary PTEC were isolated from the macroscopically ‘healthy’ portion of kidney cancer nephrectomies and stratified as normal and diseased PTEC based on the histological absence or presence of a coexisting pathology (glomerulosclerosis, arteriosclerosis, tubulointerstitial fibrosis, or tubular atrophy) within the kidney cortical tissue. We demonstrate that normal PTEC respond to oxidative stress as anticipated, with significantly increased mitochondrial dysfunction and cell necrosis. In contrast, under equivalent conditions of oxidative stress, diseased PTEC maintain mitochondrial function and cell viability. These findings are in line with the in vivo survival and accumulation of maladaptive PTEC that inhibits tubular regeneration and contributes to renal impairment after kidney injury. We propose that our novel results provide a clinically-relevant model by which to unlock the cellular and molecular pathways of oxidant-induced human kidney disease.

## 2. Results

### 2.1. Significantly Increased Levels of Oxidative Stress in Histologically ‘Diseased’ Kidney Cortical Tissue

**Figure 1:**
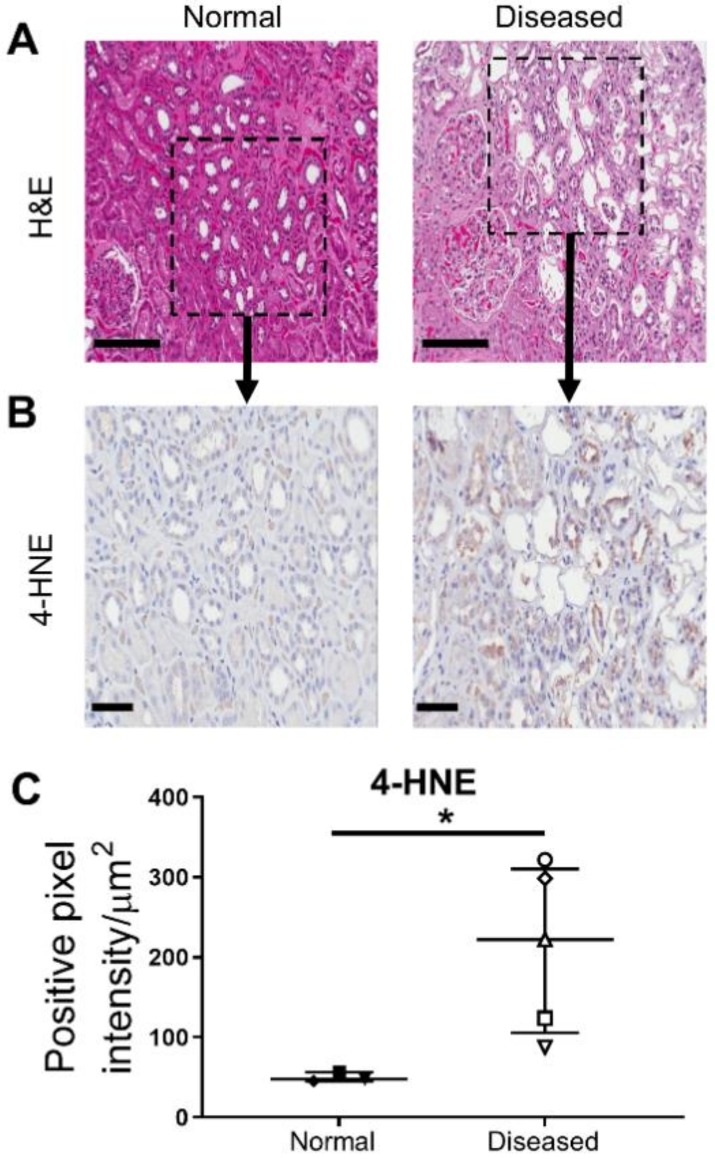
Significantly increased levels of oxidative stress in histologically ‘diseased’ cortical tissue. (**A**) Hematoxylin (purple) and eosin (pink) (H&E) staining of tissue sections from histologically ‘normal’ (left panel) and ‘diseased’ cortical tissue (right panel) under light microscopy. Representative images from one of five ‘normal’ tissue samples and one of five ‘diseased’ tissue samples. Scale bars represent 200 µm. (**B**) IHC labelling of histologically ‘normal’ (left panel) and ‘diseased’ cortical tissue (right panel) probed for 4-hydroxynonenal (4-HNE). IHC staining images from representative areas indicated by the black squares in Figure 1A are presented. Scale bars represent 60 µm. (**C**) Quantitative analysis (positive pixel intensity/µm^2^ area) of 4-HNE staining in histologically ‘normal’ and ‘diseased’ cortical tissue. Symbols represent individual donor PTEC; *n* = 3 ‘normal’ and *n* = 5 ‘diseased’ tissue samples. Horizontal bars represent medians, with interquartile range also presented. * *p* < 0.05, Mann–Whitney test.

Kidney cortical tissue was obtained from the macroscopically-healthy portion of renal cell carcinoma (RCC) nephrectomies from a total of ten donors with preserved kidney function. Specimens were grouped into histologically ‘normal’ (*n* = 5; 2 female/3 male; mean age of 58.6 ± 8.1 years; mean eGFR 81.6 ± 7.5 mL/min/1.73m^2^) or ‘diseased’ cortical tissue (*n* = 5; 3 female/2 male; mean age of 54.8 ± 3.3 years; mean eGFR 81.2 ± 8.0 mL/min/1.73m^2^) based on the absence or presence of coexisting pathology in the nontumor kidney parenchyma (Table 1 and Figure 1A). 

Amongst products of lipid peroxidation, 4-hydroxynonenal (4-HNE) is one of the most bioactive, and thus, is established as a biomarker of oxidative stress [10]. In line with the histological features of the kidney cortical tissue samples, immunohistochemical (IHC) staining demonstrated significantly elevated 4-HNE expression in ‘diseased’ specimens compared with ‘normal’ specimens (Figure 1B,C). Notably, we detected strong tubular positivity for 4-HNE in ‘diseased’ specimens, particularly localized to sites of pathological injury.

### 2.2. Normal and Diseased PTEC Display Comparable Morphological and Molecular Profiles

**Figure 2:**
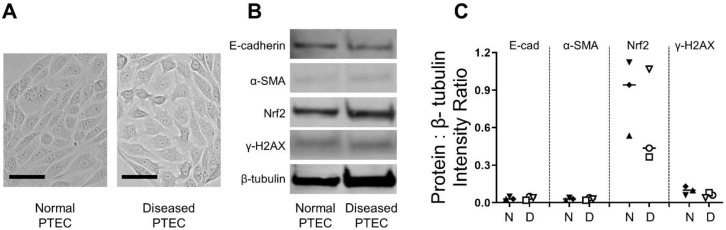
Human primary PTEC isolated and cultured from histologically normal and diseased cortical tissue display similar morphological and molecular profiles. (**A**) Light microscopy images of normal and diseased PTEC. Representative images from one of five normal PTEC and one of five diseased PTEC are shown. Scale bars represent 50 µm. (**B**) Western blot for E-cadherin, α-SMA, Nrf2, and γ-H2AX in whole cell extracts (20 µg total protein) from normal and diseased PTEC. Representative data from one of three normal PTEC and one of three diseased PTEC are presented. Full unedited gels available in the Supplementary File. (**C**) Relative expression of E-cadherin, α-SMA, Nrf2, and γ-H2AX (protein intensity as a ratio of loading control β-tubulin) in normal (N) and diseased (D) PTEC. Symbols represent individual donor PTEC; *n* = 3 donor PTEC per group.

Human primary PTEC were subsequently isolated and cultured from these specimens. Initial experiments were performed to examine the morphology and molecular signature of PTEC isolated from cortical tissue with no histological indication of renal pathology (defined as normal PTEC) in comparison to those isolated from cortical tissue with histological evidence of renal pathology (diseased PTEC). Both normal and diseased PTEC displayed characteristic cobblestone morphology (Figure 2A). Expression levels of epithelial marker E-cadherin were similar between normal and diseased PTEC, with no or low expression of mesenchymal marker α-smooth muscle actin (α-SMA) in both normal and diseased PTEC (Figure 2B,C). The expressions of nuclear factor erythroid 2-related factor 2 (Nrf2), a key regulator of the cellular antioxidant response [11], and γ-H2AX, a histone protein that contributes to chromatin-remodeling and responds to double-strand DNA breaks [12], were also comparable between the two PTEC groups (Figure 2B,C). 

### 2.3. Normal and Diseased PTEC Phenotypically Respond to H_2_O_2_-Induced Oxidative Stress

**Figure 3:**
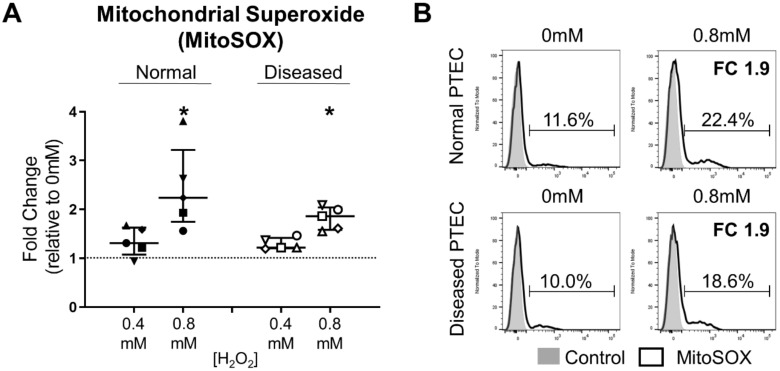
Significantly elevated mitochondrial superoxide levels in normal and diseased PTEC during high-level oxidative stress. (**A**) Fold changes (relative to 0 mM H_2_O_2_ cells) in mitochondrial superoxide levels (% MitoSOX^+^ cells) in normal and diseased PTEC cultured under low-level (0.4 mM H_2_O_2_) and high-level (0.8 mM H_2_O_2_) oxidative stress conditions. The dashed line represents a fold change of 1. Symbols represent individual donor PTEC; *n* = 5 donor PTEC per group. Horizontal bars represent medians, with interquartile range also presented. * *p* < 0.05 vs 0 mM H_2_O_2_, Friedman test with Dunn’s post-test. (**B**) Representative donor histograms of MitoSOX staining (black unfilled) in normal and diseased PTEC cultured in the absence (0mM) and presence (0.8 mM) of H_2_O_2_ compared with unstained control (grey filled). Mitochondrial superoxide levels (% MitoSOX^+^ cells) are presented for each histogram, with fold change (FC) values relative to 0 mM H_2_O_2_ cells also shown.

We next examined the phenotypic response of normal and diseased PTEC cultured in the absence (0 mM hydrogen peroxide; H_2_O_2_) and presence of low-level (0.4 mM H_2_O_2_) or high-level (0.8 mM H_2_O_2_) oxidative stress. Increased ROS production is a hallmark of oxidative stress, with superoxide the primary ROS species formed within mitochondria [13]. Baseline mitochondrial superoxide levels (% MitoSOX^+^ cells) were similar between normal and diseased PTEC under control (0 mM H_2_O_2_) conditions (Figure 3B). Both normal and diseased PTEC responded to H_2_O_2_-induced oxidative stress with an increase in mitochondrial superoxide levels (Figure 3A,B). For both PTEC groups, the fold change in MitoSOX^+^ cells during high-level oxidative stress was significantly elevated compared with control (0 mM H_2_O_2_) conditions (Figure 3A,B).

**Figure 4:**
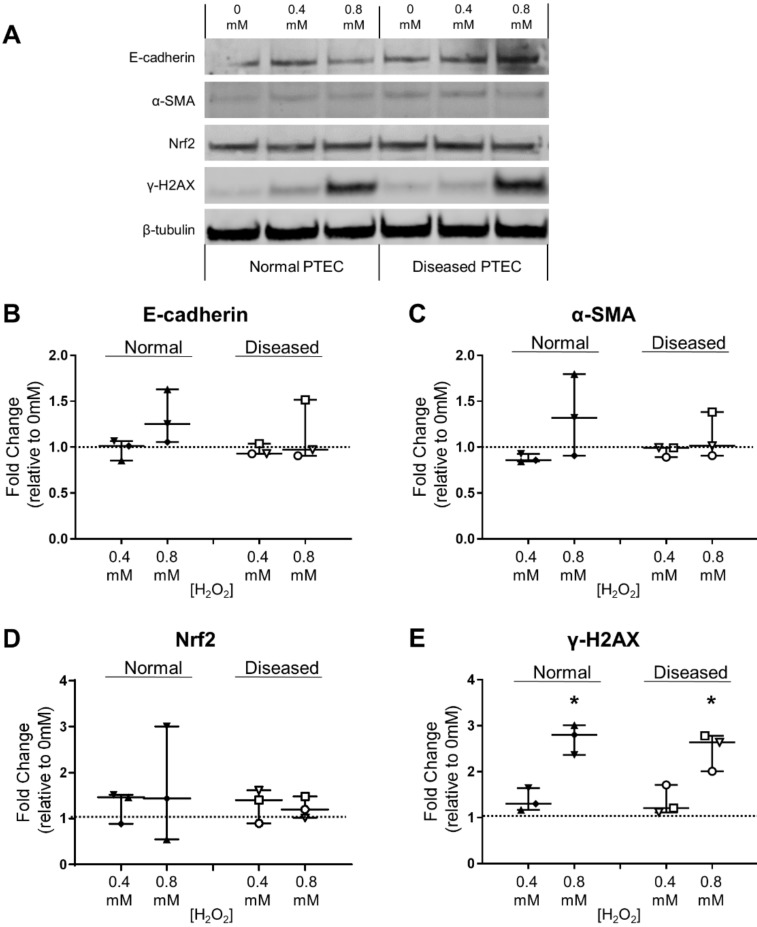
Significantly elevated DNA damage response in normal and diseased PTEC during high-level oxidative stress. (**A**) Western blot for E-cadherin, α-SMA, Nrf2 and γ-H2AX in whole cell extracts (20 µg total protein) from normal and diseased PTEC cultured in the absence (0 mM H_2_O_2_) or presence of low-level (0.4 mM) and high-level (0.8 mM) H_2_O_2_. Representative data from one of three normal PTEC and one of three diseased PTEC are presented. Full unedited gels available in the Supplementary File. (**B–E**) Fold changes (relative to 0 mM H_2_O_2_ cells) in E-cadherin (**B**), α-SMA (**C**), Nrf2 (**D**), and γ-H2AX (**E**) protein levels in normal and diseased PTEC cultured under low-level (0.4 mM H_2_O_2_) and high-level (0.8 mM H_2_O_2_) oxidative stress conditions. The dashed line represents a fold change of 1. Symbols represent individual donor PTEC; *n* = 3 donor PTEC per group. Horizontal bars represent medians, with interquartile range also presented. * *p* < 0.05 vs 0 mM H_2_O_2_, Friedman test with Dunn’s post-test.

Both normal and diseased PTEC retained expression of E-cadherin under conditions of low- and high-level oxidative stress (Figure 4A), with no significant change in E-cadherin levels observed under oxidative stress conditions compared with control conditions (Figure 4B). α-SMA and Nrf2 levels in normal and diseased PTEC were minimally affected by H_2_O_2_-induced oxidative stress (Figure 4A,C,D). In contrast, DNA damage marker γ-H2AX was significantly increased in both PTEC groups during high-level oxidative stress compared with control conditions (Figure 4A,E). These collective data confirm the capacity of both normal and diseased PTEC to respond to H_2_O_2_-induced oxidative stress.

### 2.4. Diseased PTEC Maintain Mitochondrial Function under Oxidative Stress Conditions

**Figure 5:**
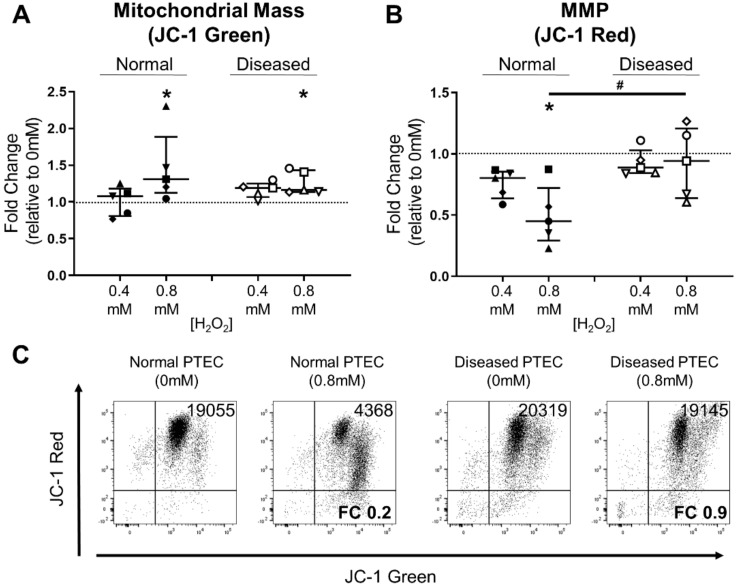
Diseased PTEC maintain mitochondrial function under high-level oxidative stress conditions. (**A,B**) Fold changes (relative to 0 mM H_2_O_2_ cells) in mitochondrial mass (JC-1 green fluorescence) (**A**) and mitochondrial membrane potential (MMP) (JC-1 red fluorescence) (**B**) in normal and diseased PTEC cultured under low-level (0.4 mM H_2_O_2_) and high-level (0.8 mM H_2_O_2_) oxidative stress conditions. The dashed line represents a fold change of 1. Symbols represent individual donor PTEC; *n* = 5 donor PTEC per group. Horizontal bars represent medians, with interquartile range also presented. * *p* < 0.05 vs 0 mM H_2_O_2_, Friedman test with Dunn’s post-test; # *p* < 0.05, Mann–Whitney test. (**C**) Representative donor JC-1 dot plots of normal and diseased PTEC cultured in the absence (0 mM) and presence (0.8 mM) of H_2_O_2_. JC-1 red delta median fluorescence intensity (MFI) values (MFI test—MFI unstained control) are presented for each dot plot, with fold change (FC) values relative to 0 mM H_2_O_2_ cells also shown.

We next examined the mitochondrial signatures of normal and diseased PTEC during oxidative stress using 5,5′,6,6′-tetrachloro-1,1′,3,3′-tetraethylbenzimi-dazolcarbocyanine iodide (JC-1) dye. This mitochondrial dye normally exists in solution as a monomer emitting a green fluorescence. However, in a reaction driven by MMP, JC-1 adopts a dimeric configuration emitting a red fluorescence [14,15]. Thus, JC-1 staining allows concurrent assessment of mitochondrial mass (green fluorescence) and MMP (red fluorescence). During high-level oxidative stress, both normal and diseased PTEC displayed significantly elevated mitochondrial mass (Figure 5A). Notably, whilst the MMP of normal PTEC was significantly attenuated during high-level oxidative stress, diseased PTEC maintained MMP levels under equivalent conditions (Figure 5B,C). These results indicate that diseased PTEC are capable of maintaining mitochondrial function, and thus, cellular health, during oxidative stress.

### 2.5. Diseased PTEC Maintain Cell Viability under Oxidative Stress Conditions

**Figure 6:**
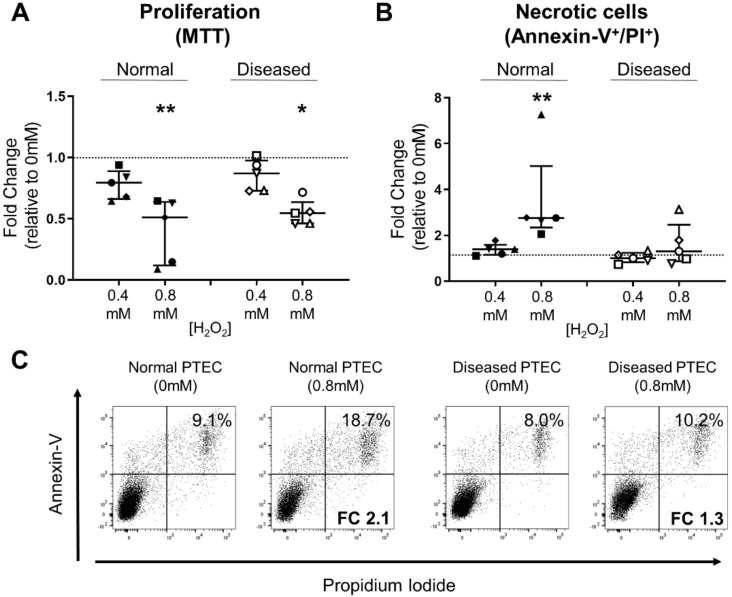
Diseased PTEC maintain cell viability under high-level oxidative stress conditions. (**A,B**) Fold changes (relative to 0 mM H_2_O_2_ cells) in cell proliferation (MTT assay) (**A**) and PTEC necrosis (% Annexin-V^+^ PI^+^ cells) (**B**) in normal and diseased PTEC cultured under low-level (0.4 mM H_2_O_2_) and high-level (0.8 mM H_2_O_2_) oxidative stress conditions. The dashed line represents a fold change of 1. Symbols represent individual donor PTEC; *n* = 5 donor PTEC per group. Horizontal bars represent medians, with interquartile range also presented. * *p* < 0.05, ** *p* < 0.01 vs 0 mM H_2_O_2_, Friedman test with Dunn’s post-test. (**C**) Representative donor Annexin-V/PI dot plots of normal and diseased PTEC cultured in the absence (0 mM) and presence (0.8 mM) of H_2_O_2_. The percentage of Annexin-V^+^ PI^+^ necrotic cells for each dot plot are presented, with fold change (FC) values relative to 0 mM H_2_O_2_ cells also shown.

We next examined the functional impact (proliferation, viability) of these differential PTEC mitochondrial signatures. Both normal and diseased PTEC displayed significantly reduced cell proliferation under conditions of high-level oxidative stress compared with control conditions (Figure 6A).

PTEC death was also assessed in this in vitro model by Annexin-V/propidium iodide (PI) staining. We detected significantly increased levels of cell necrosis (% Annexin-V^+^ PI^+^ cells) in normal PTEC during high-level oxidative stress compared with control conditions (Figure 6B,C). In contrast, diseased PTEC maintained cell viability under equivalent injurious conditions (Figure 6B,C). Collectively, these data suggest that human PTEC from diseased cortical tissue display resistance to high-level oxidative stress, which, in turn, preserves cell viability.

## 3. Discussion

The response of PTEC to injurious stimuli, including oxidative stress, plays a central role in the progression of human kidney disease [16,17]. The research implicating PTEC in disease progression comes predominantly from animal models or in vitro studies using transformed cell lines. To date, translation of this work to human primary PTEC has been hampered by limited access to kidney cortical tissue samples. Furthermore, until now, the contribution of the underlying histopathology of the cortical tissue to the functional response of human primary PTEC has not been established. Here, we demonstrate for the first time that, in contrast to human primary PTEC from histologically ‘normal’ tissue, PTEC from histologically ‘diseased’ tissue are more resistant to high-level oxidative stress, with a preservation of mitochondrial function (retention of MMP) and cell viability. 

The mitochondrial electron-transport chain is the major source of ROS (particularly the superoxide anion) during normal cellular metabolism [13]. However, the rate of mitochondrial ROS production is significantly increased under pathological conditions. This excessive generation of ROS leads to oxidative stress, ultimately inducing pathways of metabolic dysfunction and cell damage [7]. PTEC are particularly sensitive to oxidative stress due to their high metabolic demands required to reabsorb 80% of the filtrate (glucose, ions, etc.) that passes through the glomerulus [4]. We employed H_2_O_2_ to examine the intracellular PTEC response to oxidative stress in this present study, with H_2_O_2_ having been established as a common mediator of kidney disease, and also readily permeable to the plasma membrane [18]. Previous investigations have examined pathways of H_2_O_2_-induced oxidative stress in HK-2 cells, an immortalized human PTEC line [19,20,21,22,23]. Our present study is the first to demonstrate the induction of mitochondrial ROS (superoxide), and thus, oxidative stress in human primary PTEC in a model of H_2_O_2_-mediated injury. Notably, mitochondrial superoxide levels were significantly elevated in normal and diseased PTEC, confirming the capacity of both PTEC groups to respond to this injurious stimulus.

A significant consequence of oxidative stress is DNA damage. One of the earliest responses to DNA damage is the phosphorylation of the histone H2A variant H2AX on a serine which is four residues from the *C*-terminus (residue 139) to form γ-H2AX [12]. As a consequence, γ-H2AX formation has become a powerful and sensitive biomarker for the quantification of DNA damage. Here, for the first time, we use this biomarker to describe the DNA damage response of human primary PTEC to conditions of oxidative stress. In line with mitochondrial superoxide levels in our model of H_2_O_2_-mediated injury, we report significantly increased γ-H2AX in both normal and diseased PTEC during high-level oxidative stress.

DNA damage as a consequence of excessive ROS generation can lead to epithelial-to-mesenchymal transition (EMT) [24]. EMT of tubular epithelial cells is defined by loss of epithelial cell characteristics (reduced E-cadherin) and acquisition of an extracellular matrix-producing myofibroblast phenotype (elevated expression of α-SMA) [25,26,27]. EMT is a well-established mechanism in the development of tubulointerstitial fibrosis. H_2_O_2_-mediated oxidative stress has been previously shown to induce EMT in rat proximal tubular epithelial cells (NRK-52E) [28,29]. However, we did not identify the phenotypic switch characteristic of EMT induction (decreased E-cadherin/increased α-SMA) in our human model of H_2_O_2_-mediated injury for either normal or diseased PTEC.

Despite the equivalent phenotypic profiles of normal and diseased PTEC during oxidative stress, the functional responses (mitochondrial function, viability) of these two PTEC groups were dissimilar. The functional response of normal PTEC to oxidative stress paralleled the reported pathogenesis of human kidney disease. It is established in human acute kidney injury (AKI) and chronic kidney disease (CKD) that the vicious cycle between oxidative stress and mitochondrial dysfunction critically contributes to tubular cell death, and thus, loss of kidney function [30,31]. In agreement with this concept, human primary PTEC from normal cortical tissue exhibited mitochondrial dysfunction (reduced MMP) and cell necrosis in response to H_2_O_2_-induced oxidative stress.

In contrast, PTEC from diseased cortical tissue were more resistant to high-level oxidative stress, with a maintenance of both mitochondrial function and cell viability. These data are reminiscent of senescent tubular epithelial cells in diseased kidneys [32,33]. Senescent cells undergo a program of permanent growth arrest and resistance to cell death in response to injurious stimuli, including oxidative stress [34]. Senescent cells accumulate and persist in injured organs as a consequence of maladaptive repair and are increasingly recognized as drivers of disease progression [32]. Notably, the accumulation of senescent tubular epithelial cells has been associated with histopathological changes (glomerulosclerosis, tubular atrophy, interstitial fibrosis) and loss of kidney function in human renal diseases [33]. Having been isolated from an analogous in vivo histopathological and oxidative stress-rich microenvironment (see Table 1 and Figure 1), we speculate that the diseased human primary PTEC in our study are more prone to entering a maladaptive program of cellular senescence upon further injury, as evidenced by their resistance to in vitro oxidative stress. This concept may have significant physiological relevance to the pathogenic mechanisms underlying human kidney diseases. We propose that the equivalent in vivo survival and accumulation of diseased PTEC in response to repeated oxidative stressors may be pivotal in preventing tubular regeneration, and thus, amplifying the loss in kidney function observed during AKI and CKD.

These novel results have significant implications for the therapeutic use of antioxidants in the treatment of human kidney diseases. Our findings suggest that the clinical efficacy of antioxidant compounds may be hindered by maladaptive PTEC in diseased microenvironments that are inherently resistant to oxidative stressors. We instead propose the therapeutic testing of senolytic agents that selectively induce death of senescent cells and, in turn, allow proliferation and regeneration of neighbouring tubular epithelial cells following kidney damage. For instance, the repurposing of senolytic drugs currently in use in clinical oncology (e.g. ABT-263; BCL-2, BCL-XL, and BCL-w inhibitor) may be of therapeutic benefit for targeting cellular senescence in the diseased kidney [35]. Candidate drugs such as these could be screened in the in vitro oxidative stress model presented in this study to assay their efficacy as senolytic agents on human primary PTEC.

Clinical translation of our novel findings will be dependent on the identification of the intracellular signaling pathways mediating this functional resistance in diseased PTEC. Indeed, in our in vitro model system, we examined the role of Nrf2, a critical transcription factor that controls the expression of cytoprotective proteins (e.g., heme oxygenase-1; HO-1) involved in the antioxidant response [36]. Previous studies have reported a protective role for Nrf2 in preventing tubular disease progression [37,38]. Despite evidence of preexisting oxidative stress in ‘diseased’ cortical tissue (Figure 1B), we detected comparable Nrf2 expression levels between normal and diseased PTEC under baseline conditions and in response to in vitro oxidative stress. Moreover, we were unable to detect HO-1 protein by Western blot under steady-state or oxidative stress conditions in either normal or diseased PTEC (data not shown). Further functional examination of Nrf2 and other stress-activated Nrf family members (i.e., Nrf1, Nrf3) in the cellular response of human primary PTEC to oxidative stress is now required.

Future work is also essential to examine the translatability of our findings to other non-H_2_O_2_ drivers of oxidative damage and mitochondrial dysfunction in human PTEC, including high-glucose, elevated albumin, inflammation, and hypoxia [8]. Particular emphasis should be placed on the relative response of normal versus diseased PTEC to nephrotoxic medicines that interfere with mitochondrial function and lead to oxidative stress, including antimicrobials (gentamicin, tetracycline), chemotherapeutic drugs (doxorubicin, cisplatin), and other medications (nonsteroidal anti-inflammatory drugs) [39]. 

Collectively, these results provide the first comprehensive characterization of primary PTEC isolated from normal and diseased human kidneys. A deeper mechanistic understanding of the distinct functional response of PTEC from diseased microenvironments to oxidative stress will enable the development of novel therapeutics capable of targeting the accumulation of these maladaptive cells in human kidney diseases.

## 4. Materials and Methods

### 4.1. Kidney Cortical Tissue Specimens

Kidney cortical tissue was obtained with informed patient consent from the macroscopically healthy portion of RCC nephrectomies, following approval by the Royal Brisbane and Women’s Hospital Human Research Ethics Committee (Reference Number 2002/011; Approved 4 November 2016). Kidney tissue was immediately divided for: 1) fixation in formalin for IHC analysis; and 2) isolation and culture of human primary PTEC.

### 4.2. IHC Staining

Paraffin-embedded 4 µm sections were deparaffinized and rehydrated. Endogenous peroxidase activity was blocked with 1% H_2_O_2_ for 10 min. Heat-induced antigen retrieval was performed in 0.01 M Citrate Buffer (pH 6.0) for 5 min at 125 °C. Antigen retrieval was followed by a protein block with 2% bovine serum albumin (BSA) for 30 min at room temperature. Sections were probed with anti-4-hydroxynonenal (4-HNE) (Goat polyclonal IgG; Abcam, Cambridge, MA, USA) at 4 °C overnight. Tissue sections were washed and a goat horseradish peroxidase (HRP) polymer system (Biocare Medical, Pacheco, CA, USA) was applied according to the manufacturer’s instructions. Peroxidase activity was developed with ImmPACT DAB peroxidase (Vector Laboratories, Burlingame, CA, USA) for 5 min. Sections were lightly counterstained with hematoxylin and mounted using DPX Mounting Media. Quantitative analysis (positive pixel intensity/µm^2^ area) was undertaken from three randomly selected areas for each tissue sample using ImageScope (v. 12.2.2.5015, Leica Biosystems, Mt Waverley, VIC, Australia).

### 4.3. Isolation and Culture of Human Primary PTEC

PTEC were isolated from kidney cortical tissue specimens following the method of Glynne and Evans [40] and cultured in Defined Medium (DM) as previously described [41]. All PTEC were used in experiments at passage 3.

### 4.4. Induction of Oxidative Stress in Human Primary PTEC

PTEC were seeded (100,000 cells/well in DM) in 24-well flat-bottom plates to allow overnight adherence and then further cultured for 48 hours (unless otherwise specified) in fresh DM in the absence (0 mM H_2_O_2_) or presence of preoptimized low-level (0.4 mM) and high-level (0.8 mM) H_2_O_2_ to mimic oxidative stress injury. PTEC were harvested by trypsin treatment and analyzed for protein expression by Western blotting. PTEC oxidative stress, mitochondrial function and viability were assessed by flow cytometry, with cell acquisition performed on an LSR Fortessa (BD Biosciences, San Jose, CA, USA) and data analyzed with FlowJo software (TreeStar, Ashland, OR, USA).

### 4.5. Western Blotting

PTEC were lysed with Pierce™ RIPA lysis buffer (Pierce Protein Biology/Thermo Fisher Scientific, Waltham, MA, USA) containing protease inhibitor (Sigma-Aldrich, St Louis, MO, USA) and protein content determined using the BCA protein assay (Pierce Protein Biology/Thermo Fisher Scientific). Polyacrylamide gel electrophoresis (PAGE) was undertaken using standard reagents from Invitrogen (Eugene, OR, USA). Samples were denatured for 5 min at 95 °C, loaded onto Bolt™ 4–12% Bis-Tris Plus Gels, run at 200V for 26 min and transferred to a nitrocellulose membrane at 10V for 68 min. Membranes were blocked for 1 hour at room temperature using Odyssey^®^ blocking buffer (LI-COR, Lincoln, NE, USA) and subsequently probed with primary antibodies (Ab) overnight at 4 °C, including E-cadherin (Rabbit monoclonal IgG; Clone 24E10; Cell Signaling Technology, Danvers, MA, USA), α-SMA (Mouse monoclonal IgG2a; Clone 1A4; Sigma-Aldrich), Nrf2 (Rabbit polyclonal IgG; Abcam), γ-H2AX (Mouse monoclonal IgG; Clone 9F3; Abcam) and β-tubulin (Rabbit polyclonal IgG; Abcam). Proteins were visualized with IRDye 800CW goat antimouse (Millennium Science, Mulgrave, VIC, Australia) or IRDye 680LT goat antirabbit (Millennium Science) using the Odyssey CLX (LI-COR). Quantitative analysis of protein intensities relative to loading control β-tubulin was performed using Image Studio v. 2.0 software (LI-COR, Lincoln, NE, USA).

### 4.6. Mitochondrial Superoxide Detection

Mitochondrial superoxide levels were evaluated using MitoSOX™ Red (Invitrogen) [42]. Briefly, harvested PTEC were incubated with MitoSOX reagent (1 µM, 37 °C, 30 min) with mitochondrial superoxide levels (expressed as MitoSOX^+^ cells) determined by flow cytometry.

### 4.7. Assessment of Mitochondrial Changes

Mitochondrial assessments were performed using cationic dye JC-1 (Invitrogen) [14,43]. Briefly, PTEC were cultured for 24 h in the absence or presence of H_2_O_2_. PTEC were harvested and incubated with JC-1 (10 µL of 200 µM stock, 37 °C, 30 min), with JC-1 green fluorescence emission (~529 nm) and red fluorescence emission (~590 nm) detected by flow cytometry. JC-1 green fluorescence, representing the monomeric form of JC-1 and corresponding to mitochondrial mass, was calculated as the delta median fluorescence intensity (delta MFI) (MFI test—MFI unstained control). JC-1 red fluorescence, corresponding to the J-aggregate form of JC-1 and an indicator of MMP, was similarly expressed as the delta MFI.

### 4.8. Annexin V/PI Viability Assay

The Annexin-V Apoptosis Detection kit I (BD Biosciences) was used to assess PTEC viability. Briefly, harvested PTEC were incubated with Annexin V FITC and PI in binding buffer for 15 min at room temperature. The percentage of Annexin-V^+^ PI^+^ necrotic cells was determined by flow cytometry.

### 4.9. Cell Proliferation Measurements

Cell proliferation was investigated using the colorimetric MTT (3-(4,5-dimethylthiazol-2-yl)-2,5-diphenyltetrazolium bromide) Cell Proliferation Assay kit (Molecular Probes, Eugene, OR, USA). PTEC were seeded (20,000 cells/well in DM) in triplicate in 96-well flat-bottom plates to allow overnight adherence, and then further cultured for 48 h in fresh DM in the absence (0 mM H_2_O_2_) or presence of low-level (0.4 mM H_2_O_2_) and high-level (0.8 mM H_2_O_2_) oxidizing conditions. The MTT solution (10 µL of 12 mM stock) was administered to PTEC, followed by a 2.5 h incubation at 37 °C. The MTT-containing medium was subsequently removed and dimethyl sulfoxide (DMSO) (Sigma-Aldrich) applied to the cells, followed by a 10 min incubation at 37 °C. Absorbance values at 540nm were determined using a Powerwave X52 microplate reader (BioTek Instruments, Winooski, VT, USA).

### 4.10. Statistics

All data were normalized as a fold change relative to untreated control (0 mM H_2_O_2_) levels. All statistical tests were performed using Prism v. 7.0 analysis software (GraphPad Software, La Jolla, CA, USA). Comparisons between unpaired groups were performed using a Mann–Whitney test and multiple paired comparisons were performed using a Friedman test with Dunn’s post-test. *P* values ≤ 0.05 were considered statistically significant.

## Figures and Tables

**Table 1:** Clinical and histological features of patients at the time of nephrectomy.

Patient	Age (Years)/Sex (M/F)	Primary Disease	eGFR	Coexisting Pathology in Nontumor Renal Parenchyma (% Diseased Cortical Area)
Normal PTEC (*n* = 5)				
1	64/F	Clear cell RCC (Grade 2)	72	Nil
2	58/M	Clear cell RCC (Grade 2)	77	Nil
3	65/F	Clear cell RCC (Grade 2)	81	Nil
4	61/M	Clear cell RCC (Grade 2)	88	Nil
5	45/M	Clear cell RCC (Grade 2)	90	Nil
Diseased PTEC (*n* = 5)				
1	53/M	Chromophobe RCC	75	Glomerulosclerosis (5–10%)
2	56/F	Chromophobe RCC	90	Glomerulosclerosis (5–10%)
3	52/F	Clear cell RCC (Grade 2)	90	Arteriosclerosis (25–30%)
4	60/M	Clear cell RCC (Grade 4)	75	Arteriosclerosis (10–20%)
5	53/F	Chromophobe RCC	76	Tubular atrophy (10–20%)

## Supplementary Materials

Supplementary Materials can be found at https://www.mdpi.com/1422-0067/21/2/560/s1.

## Abbreviations

Ab	Antibody
AKI	Acute kidney injury
BSA	Bovine serum albumin
CKD	Chronic kidney disease
eGFR	Estimated glomerular filtration rate
DM	Defined medium
DMSO	Dimethyl sulfoxide
EMT	Epithelial-to-mesenchymal transition
H&E	Hematoxylin and eosin
4-HNE	4-Hydroxynonenal
HO-1	Heme oxygenase-1
H_2_O_2_	Hydrogen peroxide
IHC	Immunohistochemical
JC-1	5,5′,6,6′-tetrachloro-1,1′,3,3′-tetraethylbenzimi-dazolcarbocyanine iodide
MFI	Median fluorescence intensity
MMP	Mitochondrial membrane potential
MTT	3-(4,5-dimethylthiazol-2-yl)-2,5-diphenyltetrazolium bromide
NRF2	Nuclear factor erythroid 2-related factor 2
PAGE	Polyacrylamide gel electrophoresis
PI	Propidium iodide
PTEC	Proximal tubular epithelial cells
RCC	Renal cell carcinoma
ROS	Reactive oxygen species
α-SMA	α-smooth muscle actin
